# Testicular Mixed Germ Cell Tumor (TMGCT) Management: Addressing Infection and Tumor Marker Dynamics

**DOI:** 10.7759/cureus.61062

**Published:** 2024-05-25

**Authors:** Muhammad Sohail, Syed Abdullah Haider, Hamza Javed, Anusha Manazar, Mahnoor Fatimah, Zainab Wazi

**Affiliations:** 1 Urology, University of Lahore Teaching Hospital, Lahore, PAK; 2 Medicine, University College of Medicine and Dentistry, Lahore, PAK

**Keywords:** carcinoma, elevated serum tumor markers, orchidectomy, testicular germ cell tumors, tmgct

## Abstract

We report the case of a 23-year-old male presenting with right testicular swelling, post-coital pain, and fever. Initial MRI and local examination suggested testicular carcinoma. Elevated serum alpha-fetoprotein (AFP) and lactate dehydrogenase (LDH) levels were observed. Biopsy confirmed a mixed germ cell tumor (MGCT). Concurrently, the patient was diagnosed with an infection and treated with antibiotics. Remarkably, following antibiotic therapy, fever resolved, and tumor marker levels significantly decreased. Subsequent orchidectomy confirmed the diagnosis of MGCT. This case underscores the importance of recognizing and treating concurrent infections, which may influence both clinical presentation and tumor marker levels in testicular germ cell tumors.

## Introduction

Testicular mixed germ cell tumors (TMGCT) consist of two or more types of germ cell tumors, occurring in the testis. Its incidence is rising even though it is rare. WHO classified testicular germ cell tumors (TGCTs) or their precursor germ cell neoplasia in situ (GCNIS) into GCNIS-related and non-GCNIS-related tumors in 2016. GCNIS-related tumors contain cells found in the developing embryo. These are more common in men from ages 18 to 45 and are distinguished by chromosome 12p amplification while NGCNIS-related tumors comprise spermatocytic tumors, which affect older men [1]. TMGCTs show diverse histopathology, genetics, and immunocytochemistry alongside a range of clinical and prognostic outcomes, due to which it is difficult to diagnose early. The current five-year survival rate is 95% in patients who are diagnosed early while the relapse rate is between 10-20% in stage 1 seminomas [2]. The manifestation of TMGCT includes a painless lump, among which one-third show carcinoma in situ (CIS) in the testis, another one-third shows scar tissue indicative of resolved tumors, and the last one-third shows primary extra-gonadal tumors that do not affect the testicles directly. There is an elevated level of tumor markers and an increased number of chromosome i12p, a characteristic of germ cell tumors. Additionally, there may be heaviness in the scrotum, lower abdominal or groin pain, enlargement or change in shape of the testis, or accumulation of fluid in the scrotum [3]. Early diagnosis can lead to a favorable prognosis in treatment and fertility preservation, as TMGCT damages male fertility and long-term follow-up care, and helps patients and their families understand what to expect.

## Case presentation

The consent of the patient was taken before publishing this information. A 23-year-old male presented in an emergency at the University of Lahore Teaching Hospital with a complaint of testicular pain after intercourse. The pain was associated with a documented fever (39°C) that lasted for one week and right testicular swelling. Physical examination revealed erythema, warmth, and tenderness of the right scrotum with a palpable, firm mass within the right testicle.

Laboratory investigations showed elevated inflammatory markers, including C-reactive protein (3.25 mg/dl), white blood cell count (14.5 ×10^9), and neutrophils (76.1 %). The lymphocytes were decreased (12.9%) (Table 1). During the urine examination, there were abnormal findings in chemical and microscopic findings. The glomerular filtration rate (GFR) and renal function tests (RFTs) were also normal. The right testis was enlarged and markedly heterogeneous, measuring 55 x 42 × 45 mm on scrotal ultrasound. It exhibited a large central avascular hypoechoic region of 50 x 39 x 42 mm, equivalent to 44 ml, with markedly hypervascular surrounding testicular parenchyma. The epididymal head was swollen, showing heterogeneous parenchyma and markedly increased vascularity. Mild thickening and edema of the overlying skin were noted, alongside mild septated hydrocele. These features were suggestive of epididymo-orchitis with an intratesticular abscess. No abnormalities in the left testis were found.

**Table 1:** Lab results

Parameters	Patient’s results	Reference Range
C-reactive protein	3.25 mg/dL	0.8-1.0 mg/dL
White blood cell count	14.5 ×10^9/L	4.5-11.0 x 10^9/L
Neutrophils	76.1 %	40-60%
Lymphocytes	12.9 %	20-40%
Serum alpha-fetoprotein	Before antibiotics - 11.8 ng/ml; After antibiotics - 4.9 ng/ml	0 ng/mL-10 ng/mL
Serum lactate dehydrogenase	Before antibiotics - 1047 U/L; After antibiotics - 798 U/L	140-280 U/L

**Figure 1:**
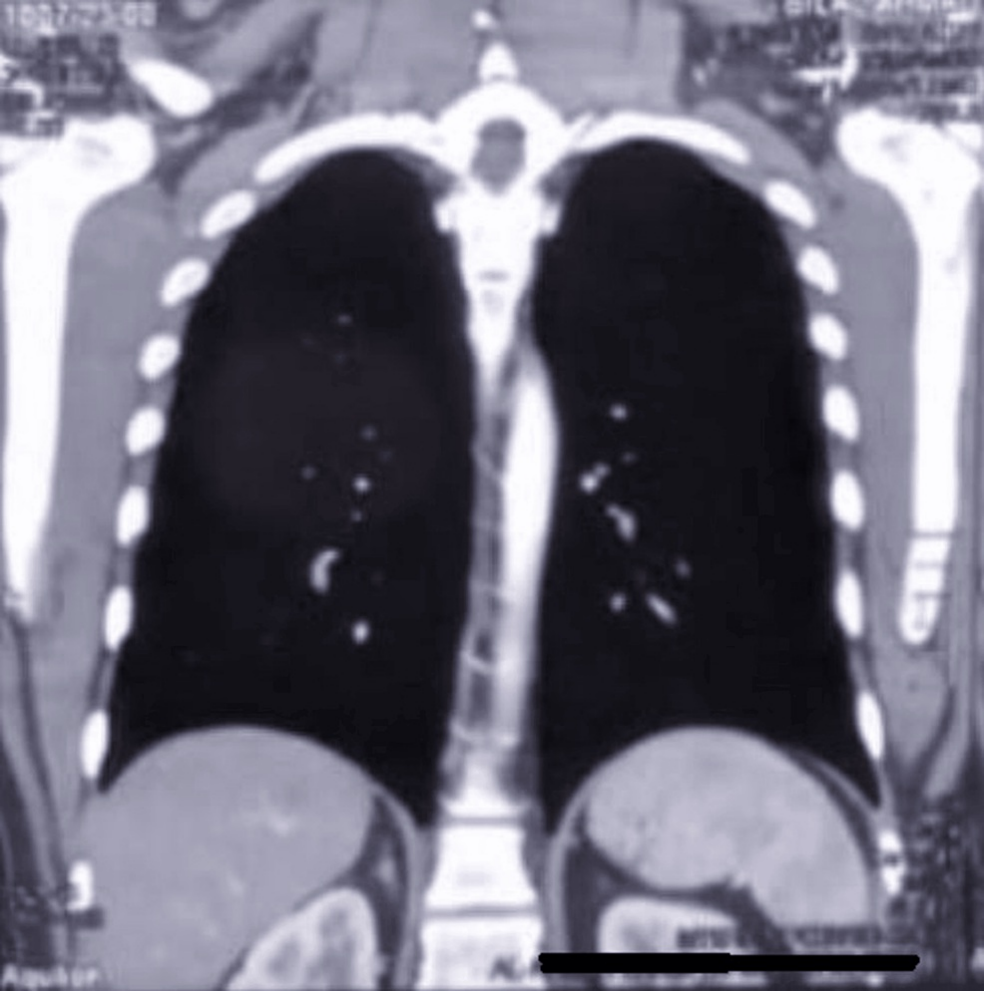
The CT scan of the chest showed no abnormalities and was deemed unremarkable

Elevated inflammatory markers and the ultrasound report indicated infection and inflammation that was accompanied by the constitutional symptom of fever. The CT chest, abdomen, and pelvis with contrast was normal. The right testis was enlarged and hypo-dense (Figure 1).

**Figure 2:**
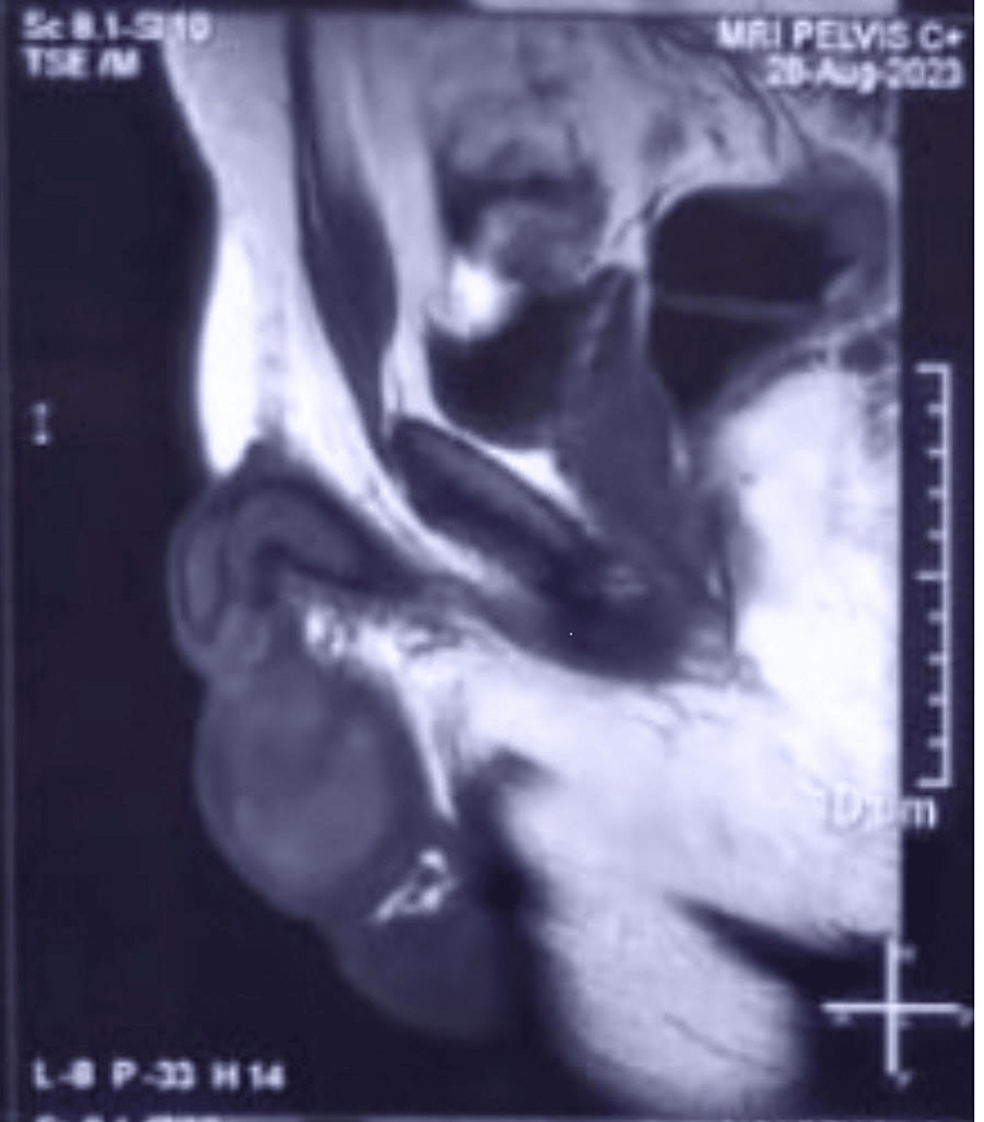
On MRI, a mass was visualized on the upper pole of the right testicle

The right scrotal Doppler ultrasound report gave the appearance of intra-testicular hematoma formation in the background of trauma. Immunohistochemical staining revealed Glypican3: weak, positive, SF1: negative, Inhibin: negative, OCT3/4: positive, CD30: positive, serum alpha-fetoprotein: 11.8 ng/ml, which was reduced to 4.9 ng/ml when the patient was treated for infection for five days, serum lactate dehydrogenase: 1047 U/L that was reduced to 798 U/L after the patient was treated for suspected infection. MRI and local examination gave a suspicion of testicular carcinoma that was resected (Figure 2).

The relation between decreasing AFP levels after treatment for suspected infection was, however, a rare phenomenon.

**Figure 3:**
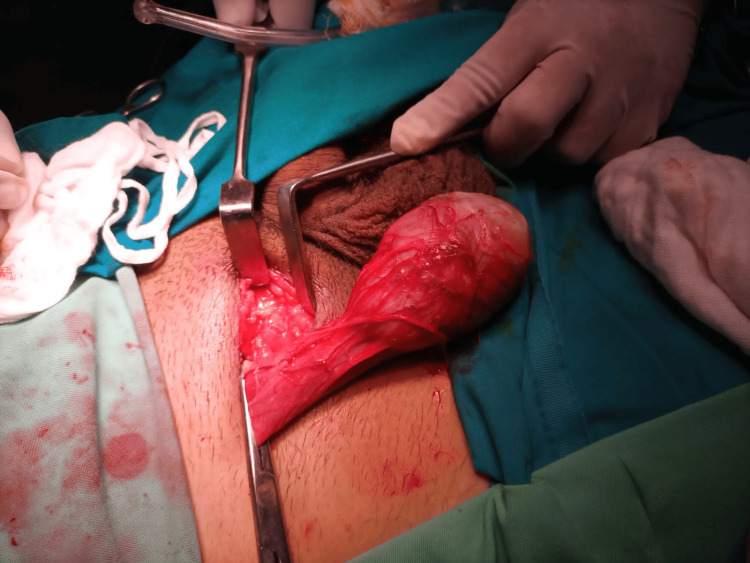
Right-sided orchidectomy being performed

A radical orchidectomy of the right testicle was done (Figure 3). The sample was sent to the laboratory and analysis showed a malignant mixed germ cell tumor, embryonal carcinoma 95%, and yolk sac tumor 5%, which was limited to the testis (PT1) and there was no involvement of the epididymis. The tumor was predominantly infarcted with few brownish viable areas with foci of necrosis. The spermatic cord and resection margin were free of tumor. No lymphovascular invasion was seen.

Initially, the epididymo-orchitis-like symptoms were treated with antibiotics and non-steroidal anti-inflammatory drugs (NSAIDs). Histological findings after right testicular orchidectomy showed a malignant mixed germ cell tumor, and the patient was sent to a tertiary care cancer hospital for chemotherapy. One cycle of the BEP regimen (Bleomycin, Etoposide, and Cisplatin) was given. Four doses were given continuously for four consecutive days and the next three doses were given with a gap of three days each. MRI and ultrasound reports showed no adverse findings and beta-HCG (beta-human chorionic gonadotropin) along with AFP levels were decreased; hence, the patient was told to follow up after six months.

## Discussion

Testicular mixed germ cell tumors (TMGCT) are the most common testicular neoplasms, comprising two or more types of germ cell tumors within the testis [1]. Germ cell neoplasia in situ (GCNIS) is a precursor lesion for TGCTs, with tumors classified as GCNIS-related or non-GCNIS-related based on their origin. TGCTs commonly affect young and middle-aged men. Early diagnosis leads to a high five-year survival rate of 95% though recurrence rates for stage 1 seminomas range from 10% to 20% [2]. These tumors are infrequent in children; nearly 1% of all solid tumors reported are of this kind. The amplification of chromosome 12p characterizes TGCTs. Spermatocytic tumors, which affect elderly men, are among the malignancies associated with non-GCNIS.

A painless lump is a common symptom of TMGCT. About one-third involve CIS in the testis, another third show scar tissue from tumor resolution, and the rest present with distinctive extragonadal tumors. Other symptoms may include testicular enlargement, scrotal heaviness, lower abdomen or groin pain, and scrotal fluid buildup. Both the quantity of chromosome i12p - a hallmark of germ cell tumors - and the level of tumor markers are enhanced [3].

It is challenging to diagnose TMGCTs early because they exhibit a wide variety of clinical and prognostic outcomes coupled with heterogeneous histology, genetics, and immunocytochemistry. There may be a higher chance of acquiring TGCTs if diagnostic radiation is exposed below the waist. Optimizing shielding techniques and minimizing medically unnecessary radiation exposure to the testicles are crucial [4].

Imaging is crucial for diagnosing, staging, planning surgery, and surveilling TGCTs. Pathologic examination ultimately confirms the diagnosis [2]. Techniques like US elastography and contrast-enhanced US help evaluate testicular lesions and differentiate between benign and malignant ones [5]. CT scans of the abdomen and pelvis detect misaligned testes, metastatic retroperitoneal adenopathy, and emergency conditions like bleeding or urinary blockage. Chest CT is recommended for late-stage TGCTs with suspected pulmonary metastases while chest radiography suffices for early-stage TGCTs [6]. MRI may distinguish seminomas from NSGCTs [7].

Urologists rely on radiological reports for histologic clues and scrotal wall involvement by the primary tumor, detecting metastases and assessing their location, size, and invasion into nearby structures, post-tumor diagnosis imaging to monitor disease progression and complications, and surveillance for recurrent and residual disease. Choriocarcinomas have a hemorrhagic component while teratomas show cystic alterations and calcifications.

The primary treatment for TMGCTs is radical orchidectomy, which involves the resection of one or both testicles with the cord as well as the internal inguinal ring. Further treatment strategies depend on tumor histology, stage, and risk factors and may include surveillance for low-risk tumors and adjuvant therapies such as cisplatin-based chemotherapy, radiation therapy, or retroperitoneal lymph node dissection. Testis-sparing surgery in selected cases with bilateral TGCTs, functionally isolated testicles, and tiny testicular masses (<2 cm) is also an option. Monitoring for cisplatin toxicity is crucial, with attention to the formation of venous or arterial thrombi and plaques in vascular structures [8].

Prognostic classification considers factors like primary tumor location (testis, retroperitoneal, mediastinal), post-orchidectomy blood tumor marker levels, and presence of nonpulmonary metastases to categorize patient prognosis as good, intermediate, or poor [9]. TGCTs are under investigation for improved diagnosis, therapy, prognosis, and surveillance using novel molecular targets. Serum or semen microRNAs, like miR-371-3, show promise for early diagnosis and surveillance of GCNIS and malignant TGCTs [10].

## Conclusions

This case underscores the importance of recognizing and managing concurrent infections in patients with testicular malignancies, specifically mixed germ cell tumors (TMGCT). The resolution of fever and decrease in tumor markers following antibiotic treatment highlights the influence of antibiotics on tumor marker levels in this case. Timely identification and treatment of both the tumor and infection are crucial for optimal outcomes. Diagnosis typically involves imaging, biopsy, and tumor marker assessment while treatment may include orchidectomy and adjuvant therapies, such as chemotherapy and radiation, depending on tumor characteristics.
